# Differently Shaped Au Nanoparticles: A Case Study on the Enhancement of the Photocatalytic Activity of Commercial TiO_2_

**DOI:** 10.3390/ma8010162

**Published:** 2014-12-31

**Authors:** Zsolt Pap, Zsejke Réka Tóth, Virginia Danciu, Lucian Baia, Gábor Kovács

**Affiliations:** 1 Faculty of Chemistry and Chemical Engineering, Babeș-Bolyai University, Arany János 11, RO-400028 Cluj-Napoca, Romania; E-Mails: tothzsejkereka@yahoo.com (Z.R.T.), vdanciu@chem.ubbcluj.ro (V.D.); 2 Faculty of Physics, Babeș-Bolyai University, M. Kogălniceanu 1, RO-400084 Cluj-Napoca, Romania; E-Mail: pap.zsolt@phys.ubbcluj.ro; 3 Research Group of Environmental Chemistry, Institute of Chemistry, University of Szeged, H-6720, Szeged, Tisza Lajos krt. 103, Hungary; 4 Institute for Interdisciplinary Research on Bio-Nano-Sciences, Babeș-Bolyai University, M. Kogălniceanu 1, RO-400084 Cluj-Napoca, Romania; 5 Faculty of Science and Informatics, Department of Applied and Environmental Chemistry, University of Szeged, H-6720, Szeged, Rerrich Béla tér 1, Hungary

**Keywords:** titanium-dioxide, gold nanoparticles, shape-control, photocatalysis, hydrogen production

## Abstract

In the present work, the influence of a gold nanoparticle’s shape was investigated on the commercially available Evonik Aeroxide P25. By the variation of specific synthesis parameters, three differently shaped Au nanoparticles were synthetized and deposited on the surface of the chosen commercial titania. The nanoparticles and their composites’ morphological and structural details were evaluated, applying different techniques such as Diffuse Reflectance Spectroscopy (DRS), X-ray Diffraction (XRD), and Transmission Electron Microscopy (TEM). The influence of the Au nanoparticles’ shape was discussed by evaluating their photocatalytic efficiency on phenol and oxalic acid degradation and by investigating the H_2_ production efficacy of the selected composites. Major differences in their photocatalytic performance depending on the shape of the deposited noble metal were evidenced.

## 1. Introduction

Since the early 1970s a large number of publications appeared regarding TiO_2_-based photocatalysts, exploring the intersection of different pathways of wastewater treatment and alternative energy sources. One of the solution for these challenges was found to be the investigation of the semiconductor-properties as well as of those related to the semiconductor–metal-based nanocomposites. In the intersection of these tasks, what can be found are the photocatalytic degradation of organic contaminants and hydrogen-production using noble-metal-modified TiO_2_.

From the time when the first report of photocatalytic water-splitting of water using TiO_2_ electrodes under UV-light appeared [1], a whole research-field has grown around the topic of designing more efficient semiconductor-based photocatalysts. Nowadays, developing a high-quality, effective, stable and cheap semiconductor-based photocatalyst for the remediation of global water pollution has become an emerging research field. In recent years, a large number of semiconductor-photocatalysts have been reported (TiO_2_ [2,3], ZnO [4,5], CdSe [6,7], SnO_2_ [8,9], CuO [10,11], WO_3_ [12,13], *etc.*), but by far, the most studied material remained the TiO_2_ because of its stability, strong oxidizing power, chemical inertness, non-toxicity, low cost and environmentally nonthreatening nature [14,15,16]. This promising material, even if, from some point of view is close to the ideal photocatalyst, it has its own “Achilles’ heels”, like limited photosensitivity for the visible light and massive recombination of photogenerated charge carriers which can decrease its catalytic efficiency.

A promising approach in the improvement of the photocatalytic activity of TiO_2_ is the deposition of noble metal nanoparticles on the semiconductor surface, which can increase the efficiency of the charge transfer process by trapping the photogenerated electrons. Among noble metals, beside Pt and Ag, gold has gained considerable attention in the enhancement of photocatalytic activity of TiO_2_. Although variously shaped nanoparticles were synthetized (e.g., cubes [17], spheres [18], nanorods [19], and triangular prisms/nanoplates [20]) for numerous (e.g., electrochemical [21], plasmonic [22], antibacterial [23], biomedical [24], catalytic [25], sensor [26]) applications, part of the above mentioned types of nanoparticles have not been tested before together with titania for photocatalytic applications.

The performances of gold deposited on the surface of TiO_2_ depends remarkably on the selected support, strong contact with the photocatalyst, Au-TiO_2_ ratio, particle size and shape. This is due to the fact that even if a large number of publication deals with the synthesis, characterization and photocatalytic applicability studies of different TiO_2_-based materials, the “legendary” P25, which is available for day-by-day use, cheap and it has generally remarkable photocatalytic property, looks to be hard to “beat”, therefore we have chosen to omit the first aspect from the present work. The second aspect, namely the contact between the nanoparticles (NP) and the photocatalyst is one of the most important factor, being observable especially in the case of gold containing mesoporous TiO_2_, where the gold made possible a more efficient charge separation of electron-hole pairs, increasing their lifetime, creating effective traps for electrons due to the formation of a Schottky-barrier at the metal–semiconductor surface, the major requirement being the intimate contact of gold with titania [27,28]. On the other hand, it was observed that in the case of the TiO_2_–Au samples prepared by photodeposition, the activity decreased with the addition of gold, probably due to the shadowing and blocking of TiO_2_ active sites by bigger and scarcely effective gold nanoparticles [29], similar effect being observed for P25–Au composites, during photocatalytic degradation of phenol under UV irradiation [30]. It has to be mentioned that generally the activity of Au–TiO_2_ composites increases as the particle size of gold decreases, reaching a maximum value of two-fold enhancement compared to pure TiO_2_, the effect observed during photodegradation of oxalic acid [31].

On the other hand, the effect of different geometries of gold nanoparticles was already summarized/studied systematically in various application, for targeted drug delivery and cancer treatment [32], therapeutical/phototermal effect [33], DNA detection [34], catalytic effect on CO oxidation [35] but to the best knowledge of the authors the effect of the various structures was not studied and correlated with their structural, photocatalytic properties and H_2_ productivity.

Thus, the main aim of this work was to investigate the impact of differently shaped gold nanoparticles deposited on the surface of commercial TiO_2_ via various methods (Diffuse Reflectance Spectroscopy (DRS), X-ray Diffraction (XRD), and Transmission Electron Microscopy (TEM)) and to correlate these with the obtained photocatalytic activity for various model organic contaminants, taking into account the intermediates’ evolution (of phenolic compounds) and H_2_ production.

## 2. Results and Discussion

### 2.1. The Proposed Research Plan

As already detailed in the introduction, the shape control of nano-sized gold is rather important, as some of the properties are exploitable only at a specific crystallite geometry. In the present work, differently shaped gold nanoparticles were obtained taking in count the dimensionality aspects, which were:

(a) Spheres: 3D particles described by a single dimension: the radius of the sphere;
(b) Triangles: 2D particles described by multiple geometric elements and for which one of the geometry defining element is significantly smaller than the other;
(c) Wires: 1D particles where two of the geometry defining element is significantly smaller than the other.

**Figure 1:**
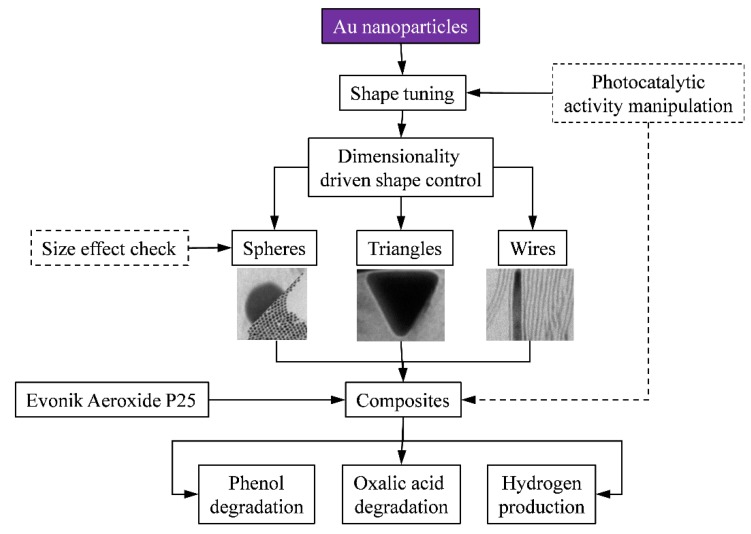
Schematic diagram of the applied research strategy.

At first sight these nanoparticles seem unrelated to each other. However, the synthesis was chosen in such way that the weight of the differently shaped gold nanoparticles were in the same range (*i.e.*, the weight of a 15 nm thick and 3 μm long nanowire is nearly the same as the weight of a 60 nm photoreduced gold nanoparticle). This was rather important as the conduction of the photogenerated electrons can occur in a different way for each type of material. To verify if this aspect is indeed true, 1 wt% Au containing Aeroxide P25 composites were obtained. Their photocatalytic activity was verified for the degradation of two different substrates (oxalic acid and phenol). Additionally, the photocatalytic hydrogen production capacity was also evaluated. The whole research plan is represented schematically in Figure 1.

### 2.2. Characterization of the Photocatalysts

#### 2.2.1. X-ray Diffraction (XRD)

**Figure 2:**
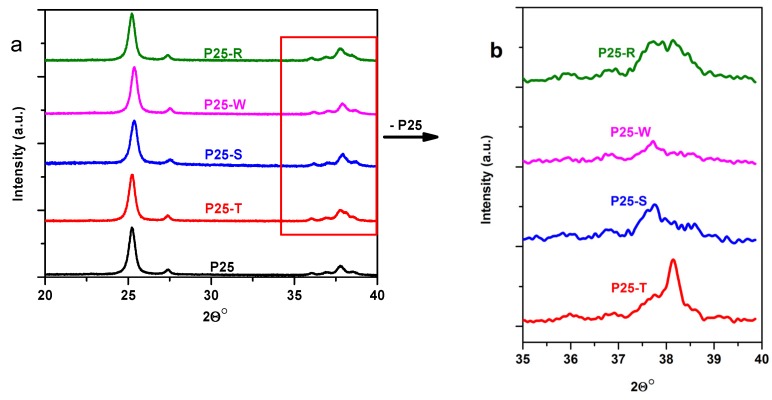
Diffraction patterns of bare and Au-modified P25 composite materials (**a**) and difference signals between P25 and the composites (**b**) (for the abbreviations of the samples, please consult Section 3.3—Synthesis of Au–TiO_2_ composites).

As a first step of characterization process, the crystal size and phase composition was evaluated using diffraction patterns. No changes were observed in these parameters of the composites comparing to P25 (Figure 2).

As expected, the X-ray diffraction patterns confirmed the particle size and crystal phase composition of P25 known from literature (89 wt% anatase, 11 wt% rutile; 25–40 nm primary particle size) [36]. Taking into consideration that the studied composite materials also contains gold it would be interesting to see if a shape dependence can be discovered in the diffraction patterns of Au. In order to analyze in-detail the diffraction patterns of Au NPs, difference diffractograms were generated by subtracting from the Au-composites’ diffraction signal the “reference” P25 diffraction pattern (in the interval between 35° and 40° (2Θ°)). The chosen 2Θ interval is of interest because the most intensive peak of Au can be found at 38° (2Θ°), for the (111) crystallographic plane [37]. The relatively narrow range, in which the diffractograms were recorded (only between 20° and 40° (2Θ°)), is explained by the fact that other diffraction peaks for Au (e.g., at 43° (2Θ°) for facet (200) or (220) at 64° (2Θ°)) are usually 3–4 times less intensive than the signal for Au facet (111) [37]. Even this mentioned signal was barely observable due to the low concentration (1%) of Au NPs in the composite materials.

Taking into account these circumstances, it can be concluded that in the case of P25-T composites the (111) facet’s signal is clearly visible at 38° (2Θ°), as this is the dominant facet in the case of triangle shaped Au nanoparticles [38]. In the case of P25-W this signal is diminished due to the less dominant presence of the mentioned crystallographic plane [39]. For the P25-S and P25-R spherical composites these signals are less visible due to the polycrystalline character of the nanoparticles. It should be noted here that the peak at 37.6° (2Θ°) is a glitch appearing due to the differentiation process.

#### 2.2.2. Transmission Electron Microscopy (TEM)

In order to have a clearer overview about the above mentioned Au nanoparticles and the P25-based composites, TEM micrographs were acquired from the “pure” colloidal Au nanoparticles and P25-based composites.

**Figure 3:**
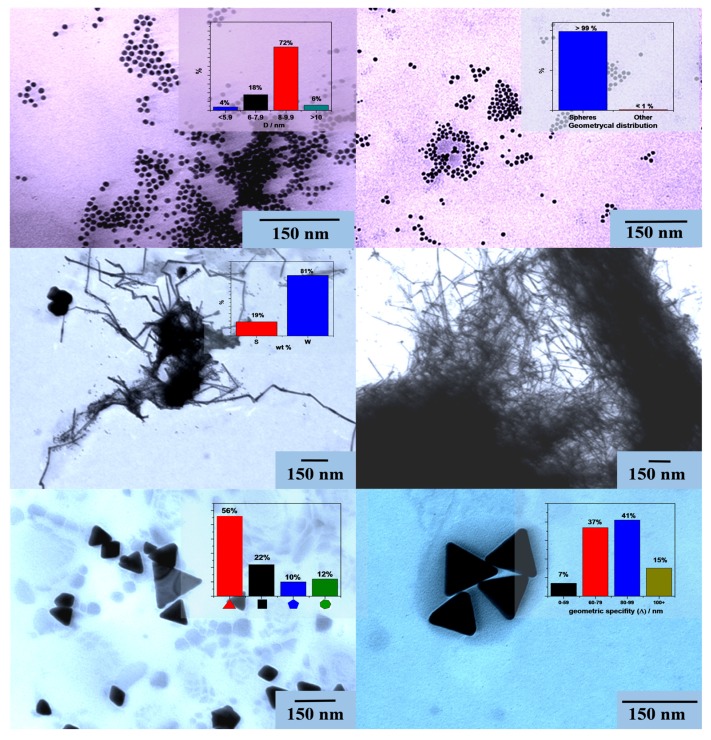
TEM micrographs of differently shaped Au-NPs (rows from up to down: spheres, wires, triangles).

The obtained micrographs have shown that the main target, namely, to obtain differently shaped Au NPs was successfully achieved as already “foretold” by the diffraction patterns. As a result of the first synthesis, relatively small and uniform Au nanospheres were obtained, with a diameter of ≈8–9.9 nm (72%). Lowering the temperature of the synthesis, as it was already described in Section 2.2 induced the formation of Au nanowires. As it can be observed in Figure 3, these nanowires had a width between 8 and 10 nm and length of ≈1–2 µm. It can be also observed that this sample, containing mostly nanowires also contained a small amount of spheres (81% *vs.* 19% weight distribution between two geometries). From the “contamination” with spheres, from the “fractures” observable on the nanowires and from the fact that the single difference of the synthesis pathways was the lower temperature in the case of nanowires, it can be concluded that the wires are “grown” from the nanospheres (Figure 3).

In order to be able to correlate the size-dependence of the Au NPs and the photocatalytic activity in the further investigations, larger spherical nanoparticles were also synthetized using the “traditional” photodeposition method, in presence of P25 (therefore, just micrograph of its composite can be shown) [40]. Using this method, we successfully reproduced the spherical Au-nanoparticles, already described, with a diameter of 50 nm. For the third sample it was observable that the synthesis of nano-triangles was successful (equilateral triangles were obtained with size measuring between 60 and 100 nm, 78%). Besides triangles smaller amounts of cubes and rhombs (22%) were detected, probably from the simultaneous twin-growth of two triangles, pentagonal (10%) and polyhedral (12%) particles.

TEM micrographs were taken also from TiO_2_–Au-based nanocomposites. On this micrographs the Au NPs can be once more observed, this time in their composite form, alongside the titania photocatalyst. Analyzing in-detail these connections between Au and TiO_2_ nanostructures and the morpho-structural particularities of the Au NPs, working mechanisms were proposed in order to make predictions for the photocatalytic activities of the composites. In the first case, when smaller spherical NPs (case of P25-S) were introduced in the composites, the generated electron-hole pairs can be separated more efficiently if the particle size is sufficiently low. This is possible due to the higher ratio of Au nanoparticles/TiO_2_ nanocrystallites which favors the efficient charge separation mechanism. Thus, as more NPs are loaded on the surface of the photocatalyst, this behavior being showed in the literature as well in previous studies [41,42]: In the case of P25-R composite the photocatalytic activity is supposed to be lower because the size of the spherical particles are higher, around 60 nm.

**Figure 4:**
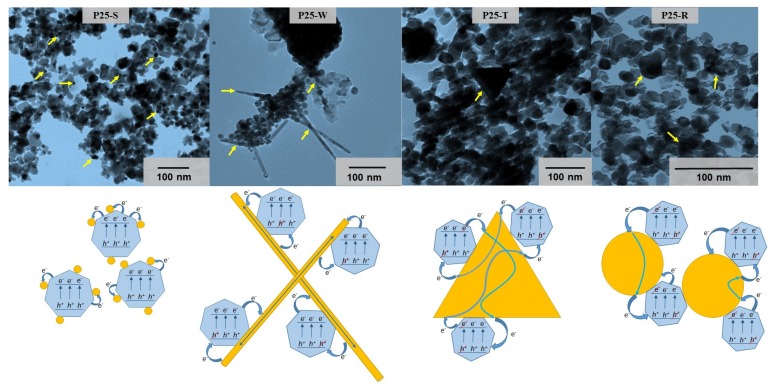
TEM micrographs and schematic representation of P25-Au composites (P25-S, P25-W, P25-T and P25-R).

For the P25-W composite the situation differs completely, observing the connection between multiple P25 particles and the lengthiness of the Au NPs (around 1 µM) that the multiple electrons are “taken” away by the nanowires from the e^−^/h^+^-pairs, but also a higher recombination rate is hypothetically possible due to the high possibility to “meet” a new TiO_2_ particle causing “nano-short-circuiting”. Similarly, a lower photocatalytic rate can occur when larger triangles build up the composites (P25-T), where more P25 particles are attached to the same Au particle. This means that the photogenerated electrons are separated efficiently in the first instance, however, there is an increased probability for the recombination process due to the high TiO_2_/Au triangle ratio (Figure 4). The theories describing the mechanisms of charge-carriers listed above were “tested” on the photocatalytic measurements using phenol and oxalic acid as model-contaminants.

#### 2.2.3. Diffuse Reflectance Spectroscopy (DRS)

The next critical parameter, from the point of view of the photocatalytic activity, was investigated, namely, the light-absorption properties of the Au NP-based composites.

The obtained Au NPs size and shape was also observable in reflectance spectra due to the surface plasmon resonances visible between 520 and 580 nm [43], showing a substantial, shape and size dependent difference in this region. It can be observed that the less intense plasmonic bands, also shifted slightly to lower wavelengths can be observed in the case of P25-S and P25-R, while samples containing larger Au nanoparticles (P25-W and P25-T) have their characteristic plasmonic band at higher wavelengths. This can be reasonably attributed to the fact that for the composites with larger nanoparticles the absorption band broadens extending to the all visible range, whereas for smaller Au NPs, the band intensity decreases, becoming close to “flat” in the case of composite with spherical gold.

**Figure 5:**
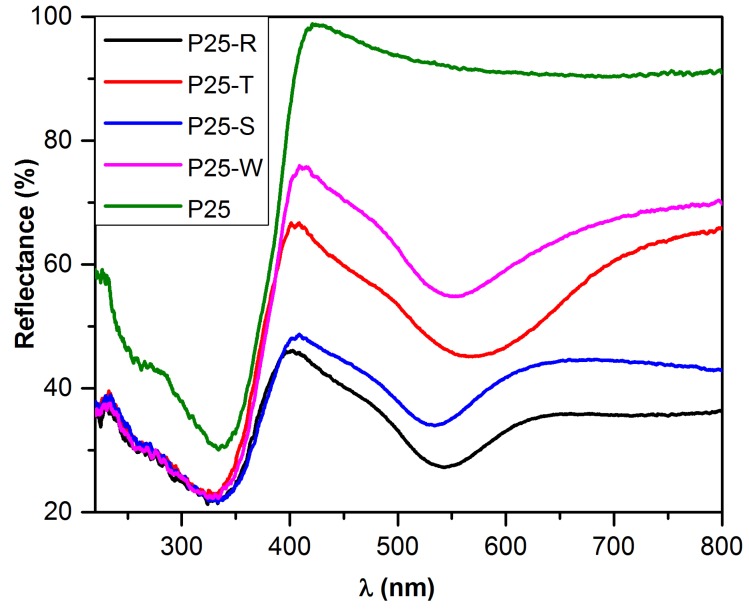
Comparison of different DRS spectra containing differently shaped Au NPs.

As it can be seen in Figure 5, the deposition of differently shaped Au particles induced significant changes in the optical properties of P25; consequently, the evaluation of the band-gap values was mandatory. Using the Kubelka-Munk equation, it was found that lowering the band-gap value by modifying the pure P25 (3.11 eV) with noble-metal was successful, the lowest value being associated with P25-S (2.70 eV), with a slightly higher value for P25-R (2.80 eV), while P25-W and P25-T had a band-gap value of 2.85 eV. This means that the alteration of the Au-shape can help for a fine tuning of the optical properties, enables a careful design of a photocatalyst with a desired band-gap.

**Figure 6:**
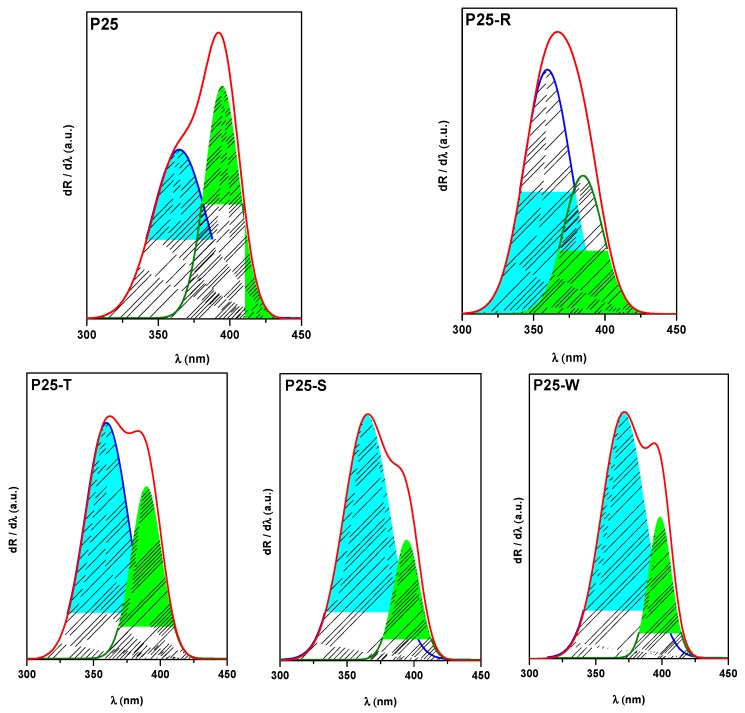
First order derivative spectra of P25-based nanocomposites.

Analyzing the first order derivative of the reflectance’s of the composites and the pure P25 itself, two electronic transitions can be observed, both being responsible for the UV light absorption of the catalysts, at ≈365 and ≈400 nm [44,45]. These peaks in terms of energy, are corresponding to band-gap values of anatase and rutile. In addition to the qualitative presence of these transitions, it is clearly visible that the addition of differently shaped Au NPs to the commercial titania induces a change in the ratio of these peaks. It can be observed in the first order derivative spectra of the composites that the ratio of anatase/rutile transitions is inverted (1/1.25 for P25 shifts to ratios around 1/0.33 and 1/0.75 in the case of composites). This behavior could be explained with the fact that the deposited gold NPs are taking the photogenerated electrons from the anatase, giving less importance to the presence of the rutile phase in the composite. The lowest A/R electron transition ratio is present in the sample P25-S, this can be attributed to the smaller Au particle’s size which can take even more electrons from the charge separation process. It can be also observed that not only the ratio, but also the position of the electron transition band, is affected by the deposited Au nanoparticles. While the position of the peak is quite similar in the case of P25 and P25-S (365 and 395 nm), the addition of large triangular shaped nanoparticles shifts both transition to higher energy value/lower wavelengths (360 and 390 nm). The P25-W composite exhibits a change in the “other direction”, the peaks can be found at higher wavelengths, at 370 and 400 nm (Figure 6).

### 2.3. Photocatalytic Performance of the Obtained Nanocomposites

In the previous section, we have shown that the shape of a noble metal nanoparticle influences greatly the composite’s overall band-gap. Thus, a series of photocatalytic measurements were performed in order to evaluate the activity differences, which could be caused by the shape and dimension of the Au-nanoparticles.

#### 2.3.1. Photodegradation of Phenol

The photodegradation of phenol was carried out under UV irradiation (λ_max_ ≈ 365 nm). UV irradiation was chosen in order to distinguish the effect of the charge separation efficiency of the gold NPs, phenomenon which is more efficient using UV irradiation, carefully eliminating the effect of any plasmon-resonance-based optical interaction (e.g., excitation, *etc.*). Our experiments regarding gold deposited titanias showed that the presence of the gold nanoparticles are inhibiting the phenol decomposition rates, fact was already was observed in our previous works [31], but they are maintaining a comparable efficiency in terms of degradation rate (50.5%–71% in 2 h, comparing to the 82.4% of P25). It is already known that for the photocatalytic decomposition of phenol the OH∙ radicals are responsible. Therefore, their generation rate is a crucial aspect, which is mainly determined by the charge separation efficiency and the nature of the substrate (adsorbing/non-adsorbing). As it was already shown in Figure 4, the gold NPs in some cases can inhibit or enhance the charge carrier separation process, depending on:

(i) The particle’s size in the base catalyst—in the present study this was ruled out by using Aeroxide P25 in all the composite materials;
(ii) Au NPs crystal size—all the Au NPs obtained are large Au NPs (except for P25-S).

In our case the larger gold NPs enhances the charge recombination rate (as discussed in Section 2.2.2), by conducting the electrons from a TiO_2_ particle to another one. Hence, the possibility to obtain photo-generated charge carriers was lowered, thus inhibiting OH∙ generation radical as well (this is why all the Au containing catalyst have lower activity towards phenol). However, not all the Au geometries favor the recombination in the same way, as was already proposed in Figure 4.

It would seem that the most “detrimental” recombination mechanism is the one described for the sample P25-T (50.5% phenol degradation yield). The “triangle” shaped Au nanoparticles are interconnecting several TiO_2_ crystals, resulting an interparticular charge neutralization, inhibiting significantly the possibility to generate reactive OH radicals. Surprisingly, small sized spherical Au NPs were also not effective in the degradation of phenol (P25-S, 52.9% degraded phenol). The latter one could mean that the degradation intermediates are seriously intervening in the degradation mechanism as already demonstrated in our recent work [44,45], although the charge separation mechanism is considered to be efficient at this Au particle size [31]. A significant inhibition of the phenol removal was also registered in the case of P25-R (58.4% of removed phenol), where the charge separation scenario was considered similar to the one proposed for P25-T. The main difference was in the electron transfer rate, which is dependent on the crystal geometry—a critical issue that is currently under investigation. The least inhibitive effect on the phenol-degradation was observed in the case of P25-W (71% of degraded phenol). Thus, the Au nanowire’s (NW) charge separation is the most effective, which should be reflected also in the oxalic acid degradation.

**Figure 7:**
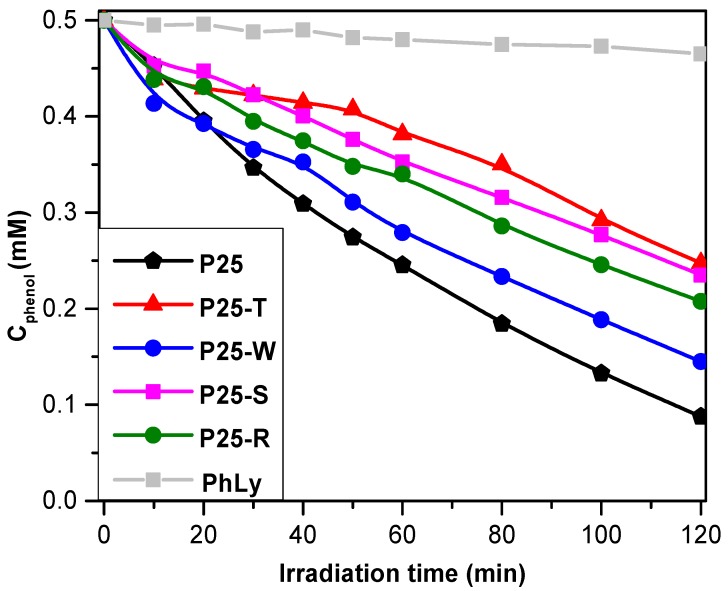
Phenol degradation curves of the prepared nanocomposites under UV irradiation (PhLy refers to the degradation of phenol without photocatalyst).

In terms of the reaction rate, the situation changes. The best performing composite was the P25-W, reaching a 7.45 × 10^−3^ mM·min^−1^ (value calculated from the first five points of the degradation process), which was higher than the pure industrial titania. The difference in the terms of efficiency measured in the degradation yield and reaction rates resides in the fact that Au NPs are playing a crucial role in the accumulation of hydroxylated intermediates. These compounds are usually detrimental for the photocatalytic efficiency [44,45]. Consequently, in the beginning of the degradation process the phenol is degraded efficiently on P25-W (even more efficient then the bare P25). As the degradation intermediates are accumulating, the phenol degradation is inhibited and the degradation kinetics are slowly changing (the shape of the degradation curve P25-W, which intersects the curve of P25—Figure 7). As it was already shown in Figure 4, the gold NPs in some cases can inhibit or enhance the charge carrier separation process, depending on the particle’s size of the base catalyst and Au NPs as well. In our case, the larger gold NPs enhances the charge recombination rate, by conducting the electrons from a TiO_2_ particle to another one. Hence, the possibility to obtain photo-generated charge carriers was lowered, thus inhibiting OH∙ generation radical as well (this is why all the Au containing catalyst have lower activity towards phenol). The other composites showed a somewhat lower photocatalytic performances: P25-T and P25-R (5.71 × 10^−3^ mM·min^−1^, 5.01 × 10^−3^ mM·min^−1^ and 4.49 × 10^−3^ mM·min^−1^, respectively). The less efficient catalyst was the P25-S, its reaction rate was lower by 40% than the best performing P25-W (Figure 7 and Table 1).

It has to be mentioned, that the photolysis under UV irradiation was ≈5.6%, value smaller with approximately one order of magnitude, compared to the results obtained using the P25-Au composites.

**Table 1:** Photocatalytic efficiencies of P25-based composites in different photocatalytic applications.

Sample	*r*_0,oxalic acid_ (mM·min^−1^) ×(10^−3^)	*r*_0, phenol_ (mM·min^−1^) ×(10^−3^)	Oxalic acid_UV/Vis_ degradation rate (%)	Phenol_UV/Vis_ degradation rate (%)	H_2_ production rate (mL·h^−1^)	Band gap (eV)
P25-W	46.0	7.45	97.4/21.0	71.0/12.2	0.62	2.85
P25-S	71.7	3.19	97.6/20.8	52.9/8.6	0.78	2.70
P25-R	51.2	4.49	86.2/19.1	58.4/9.5	0.63	2.80
P25-T	69.0	5.01	75.5/16.8	50.5/7	–	2.85
P25	41.9	5.71	54.3/12	82.4/13.8	–	3.11

As it was predictable, the less efficient composite for photodegradation was P25-T due to the larger Au NPs size and to the fact that more titania particles can be attached on the same noble metal particle, making the charge-separation less efficient. Similar process can explain the lower efficiency in the case of P25-W (described in detail at last part of Section 2.2.2).

It has to be mentioned, that photocatalytic tests were made in order to determine the efficiencies of the composites under visible light irradiation (Table 1). It can be concluded that the tendencies are similar regarding the degradation rates, but the efficiencies are generally more than 5 times smaller than those observed in UV. Therefore, due to the low degradation rates and the possible errors of measurements, the results were no longer discussed (see Supplementary Materials).

#### 2.3.2. Photodegradation of Oxalic Acid and Photocatalytic Hydrogen Production

In order to have information about the photodegradative properties of P25-Au composites in removal of non-aromatic, simple organic compounds, photodegradation measurements were carried out on oxalic acid as model contaminant, which is a well-known, good absorbing organic substrate.

**Figure 8:**
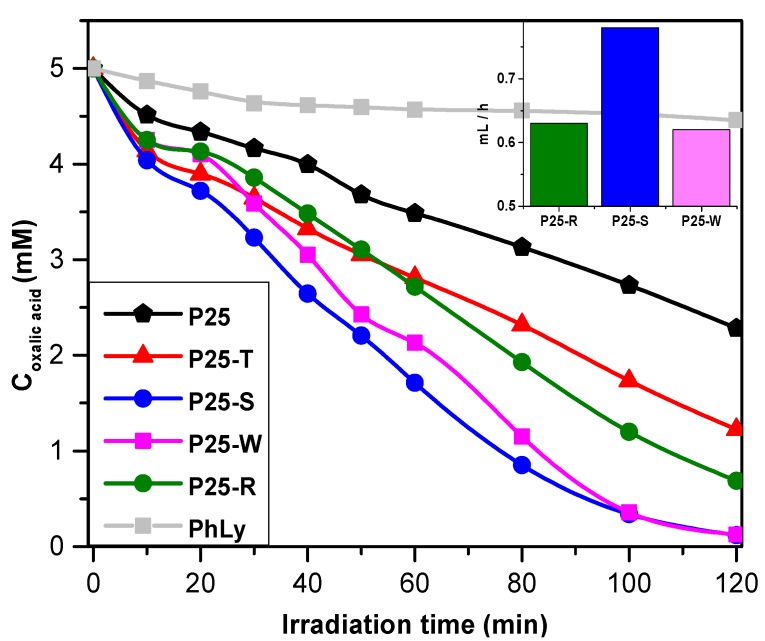
Photodegradation curves of oxalic acid in the presence of the prepared nanocomposites under UV irradiation and (inset) hydrogen production rates of the selected composites.

As it can be seen on Figure 8, all the composites have shown an increased efficiency compared to P25 due to the fact that the oxalic acid molecule is adsorbing on the surface of the catalyst, trapping in this way more efficiently the photogenerated holes (that is also the reason why oxalic acid is used as a hole-scavenger molecule in photocatalytic measurements and H_2_-production). Speaking in term of reaction rates, the best performing catalyst was the P25-S, achieving a reaction rate of 71.7 × 10^−3^ mM/min, followed by the P25-T, P25-R. The least efficient composite was the one made with Au nanowire nanoparticles, having a ≈40% lower efficiency lower with but still higher than the value reached by pure P25.

In term of conversion after the 2 h experiment, the “ranking” slightly changes. The P25-S is showing the highest conversion rate but the nanowire containing composite is also converting similar amount of oxalic acid in the range of experimental error. The composite made with photodeposition, the conversion rate is still at a high rate, for P25-T this value is decreasing to 75.5%, which is still higher with ≈30% than the value obtained using pure P25.

Photocatalytic tests were made also under visible irradiation, in order to determine the efficiencies of the composites (Table 1). Briefly, it can be concluded that the tendencies are similar regarding the degradation rates, but the efficiencies are generally around four times smaller than those observed in UV, if we are taken under the loupe the degradation rates. Therefore, due to the low degradation rates and the possible errors of measurements, the results were no longer discussed (see Supplementary Materials).

As it was already shown in the previous sections, the shape of the Au nanoparticles is vital in the definition of the photocatalytic activity of a given composite material. Hydrogen production measurements were carried out, in order to find out if the shape of the Au nanoparticles’ is indeed a determining factor in this case.

In order to determine the hydrogen production rate, oxalic acid (50 mM) was used for photocatalytic decomposition in oxygen-free conditions. The H_2_ production rate was calculated using data collected from the interval between 30 and 90 min, a time when the productivity of composites has reached a quasi-stationery rate. As it is shown in Table 1 and in Figure 8 (inset), the highest hydrogen production rate was achieved by the composite containing small spherical Au nanoparticles, fact expected looking at the results obtained by photodegradation of oxalic acid. The wire-shaped Au NPs and the NPs obtained by photodeposition had shown a lower activity, producing ≈0.62 mL·h^−1^, presenting a lower difference between their efficiencies as expected from efficacy toward oxalic acid (see also Supplementary Materials). P25-T was omitted due to its relatively low activity toward oxalic acid, shown in previous section and pure P25 was already shown inactive in previous studies in H_2_ production studies.

## 3. Experimental Section 

### 3.1. Materials

All chemicals used were of analytical grade. Trisodium-citrate was purchased from Alfa-Aesar (Karlsruhe, Germany) while the gold precursor (HAuCl_4_∙3H_2_O), oleylamine and cetyltrimethylammonium bromide (CTAB) were purchased from Sigma-Aldrich (Steinheim, Germany). Evonik Aeroxide P25 (Essen, Germany) was used, as received, without further purification for the preparation of the composite materials.

### 3.2. The Synthesis of the Au-NPs

According to Bernardi *et al.* [39] gold *nanowires* were synthetized dissolving 100 mg of HAuCl_4_ in 1 mL of ethanol. The ethanolic solution of the precursor was added to 25 mL of oleylamine preheated at 75 °C. The mixture’s temperature was kept for 6 h in a nitrogen atmosphere (without stirring). During the synthesis the color of the solution was changed from transparent to orange trough purple. Finally, a red precipitate was obtained. After 6 h the reaction product was centrifuged and washed with ethanol (with 3 × 25 mL, 1 × 16 min and 2 × 8 min, all at 4400 rpm) and with methanol (8 min, 4400 rpm). The *nanospheres* were obtained in a similar way, the only difference was that the oleylamine was heated and kept at 85 °C.

The gold *nanotriangles* were synthetized according to Kumar *et al.* [38] using two solutions: the first contained 896 µL Na_3_C_6_H_5_O_7_ (0.1 M) and 52.5 mL H_2_O while the second solution contained 31.57 mL H_2_O with 112 mg CTAB and 3.43 mL HAuCl_4_ (12.7 mM). The two solutions were added instantaneously into a pre-heated vessel (at 90 °C, without stirring) [38].

### 3.3. Synthesis of Au–TiO_2_ Composites

As it was already shown in representative publications [46], the highest photocatalytic activity of Au–TiO_2_-based composites can be obtained at ≈0.5–1 wt% gold concentration, and due to the fact that the 1 wt% of Au nanoparticles can be efficiently detected by means of the optical spectroscopy and other methods [45,48], therefore all the P25-based composites synthetized below contained 1 wt% concentration of Au NPs. Applying the above described Au synthesis pathways the TiO_2_–Au composites, based on commercial titania, were prepared as follows:

P25-W was made adding 6.474 mL of Au nanowires suspension (freshly synthetized and dispersed in 100 mL of hexane) to a P25 suspension (made from 800 mg P25 and 50 mL of hexane). In the final step, the suspension was dried for 24 h at 40 °C;

P25-S was prepared using 4 mL of spherical Au-sol added to a suspended P25 (800 mg in 150 mL) under vigorous stirring. The obtained mixture was dried for 24 h, washed with acetone (3 × 50 mL) and dried once more for 24 h;

P25-T was prepared similar to P25-S, adding 83.63 mL of Au(t) sol in 800 mg/150 mL (H_2_O) P25 suspension, washed with water, dried for 24 h, washed three times (with H_2_O) and dried once more for 24 h;

P25-R using the method of *photoreduction*, namely mixing Au-precursor (3.22 mL, 12.7 mM) in 200 mL, 50 mM oxalic acid solution with P25 (4 g/L). The mixture was kept under vigorous stirring, irradiated with UV light (3 × 60 W, λ_max_ ≈ 365) for 5 h and washed similarly to P25-T_._

To ensure about the fact that all amount of Au NPs was impregnated successfully on the surface of the TiO_2_, obtaining in this way the optimal 1 wt% concentration of gold in the composites, after the centrifugation spectrophotometric measurements were made using the supernatant. On the adsorption spectra’s no specific plasmonic resonances were observed, fact which was also observable with “naked eye”, the supernatant being clear/colorless.

### 3.4. Characterization Methods and Instrumentation

X-ray diffraction (XRD) measurements were performed on a Rigaku diffractometer (Prague, Czech Republic), λ_CuKα_ = 0.15406 nm, 40 kV, 30 mA, in the 20°–40° (2Θ) regime). The average diameter of the particles were estimated using the Scherrer equation. The weight fraction of anatase and rutile was calculated for each sample from the peak areas of the anatase and rutile peaks at 25.3° (2Θ) and 27.5° (2Θ), respectively, using the relationship developed by Zhang and Banfield [47], and the crystallites average size was calculated using the Scherrer equation [48].

Transmission electron microscopic (TEM) measurements were executed to characterize the particle size and to identify the morphology of the particles. The TEM micrographs were recorded on a Philips CM 10 instrument (Amsterdam, Netherlands) operating at 100 kV using Formvar coated copper grids.

JASCO-V650 spectrophotometer (Tokio, Japan) with an integration sphere (ILV-724) was used for measuring the DRS spectra of the samples (λ = 250–800 nm). To obtain the band-gap energy the reflectance data were converted to F*(R)* values according to the Kubelka-Munk theory. The band gap was obtained from the plot of [F*(R)*·*E*]^1/2^ *versus* energy of the exciting light (*E*).

The hydrogen production experiments were executed in a Pyrex glass photoreactor (Szeged, Hungary) with 170 mL reactor volume, filled with 150 mL suspension) thermostated at 25 °C and surrounded by 10 × 15 W fluorescent, low pressure mercury lamps (λ_max_ ≈ 365 nm). The suspension’s concentration was 1.0 g/L and the applied sacrificial agent was oxalic acid (50 mM). During the photocatalytic runs the suspension was continuously purged with N_2_ (50 mL/min) to avoid the presence of O_2_. The H_2_ gas evolved was determined with a Hewlett-Packard 5890 type gas chromatograph (New York, NY, USA) equipped with a thermal conductivity detector, using a 2 mL sampling loop (sampling times: in the first hour 10 min, in the second hour 20 min). On the basis of the H_2_ concentrations determined by gascromatography from the flow rate of the N_2_, the rate of H_2_-evolution (*r*) at the time of the sampling has been determined. The total amount of produced hydrogen was estimated by integrating the area under the hydrogen evolution curve. The duration of the experiment was 2 h.

The performance of the catalysts prepared was characterized by using the photocatalytic decomposition of phenol in solution using an open tube, double walled photoreactor (100 mL), surrounded by a thermostating jacket (25 °C). The continuously stirred reactor was surrounded and irradiated by six fluorescent lamps). The continuously stirred reactor was surrounded and irradiated by six fluorescent lamps (6 W power, λ_max_ ≈ 365 nm). The Vis photoreactor (400 mL, *c*_P25-Au_ =1.0 g·L^−1^) is an immersion type HERAEUS Reactor system equipped with an OSRAM metal halide lamp (Power Star HCl-TC 75W/WDL type, Istanbul, Turkey) with a NaNO_2_ solution (1 M) in a thermostating jacket absorbing UV photons (λ < 400 nm) [30]. The concentration of phenol and oxalic acid was measured with an High Performance Liquid Chromatography consisting of a Merck-Hitachi L-7100 low-pressure gradient pump (Tokio, Japan) equipped with a Merck-Hitachi L-4250 UV–vis detector and a Lichrospher RP 18 column applying methanol/water mixture and H_2_SO_4_, respectively as eluent. Detection wavelength was set at the lower wavelength absorption maximum of phenol (*i.e.*, λ = 210 nm), the high molar absorption coefficient of which makes the detection of small quantities of the substrate possible. 

## 4. Conclusions 

The present work shows a systematic overview concerning the importance of the shape of Au nanoparticles in photocatalytic applications. It was shown that by adjusting the reaction parameters during Au NPs production, obtaining differently shaped particles and introducing them in composites can change considerably the optical properties of the materials, including the electron transition bands.

Additionally, the activity was also dependent on the shape of the Au nanoparticles. Generally; it can be concluded that in the case of phenol, the addition of Au NPs can decrease the efficiency of commercial catalyst, while for a good adsorbing substrate, the efficiency can be increased by ≈100% (degradation rate obtained at the end of 2 h measurement). For hydrogen production, the shape-dependence was also proved, e.g., the differences occurring for oxalic acid degradation in the case of composites containing Au nanowires and nanotriangles were diminished.

It can largely be concluded that the shape of the deposited nanoparticles can highly influence the efficiency of the composites, making possible a shape defined application tuning.

## Supplementary Materials

Supplementary File 1
