# Supplementary material for: Differently Shaped Au Nanoparticles: A Case Study on the Enhancement of the Photocatalytic Activity of Commercial TiO2
Source: Materials (Basel). 2014 Dec 31;8(1):162–80. doi: 10.3390/ma8010162 (PMC5455219; doi:10.3390/ma8010162)
Supplement: Supplementary File 1 [file materials-08-00162-s001.pdf]

Supplementary materials

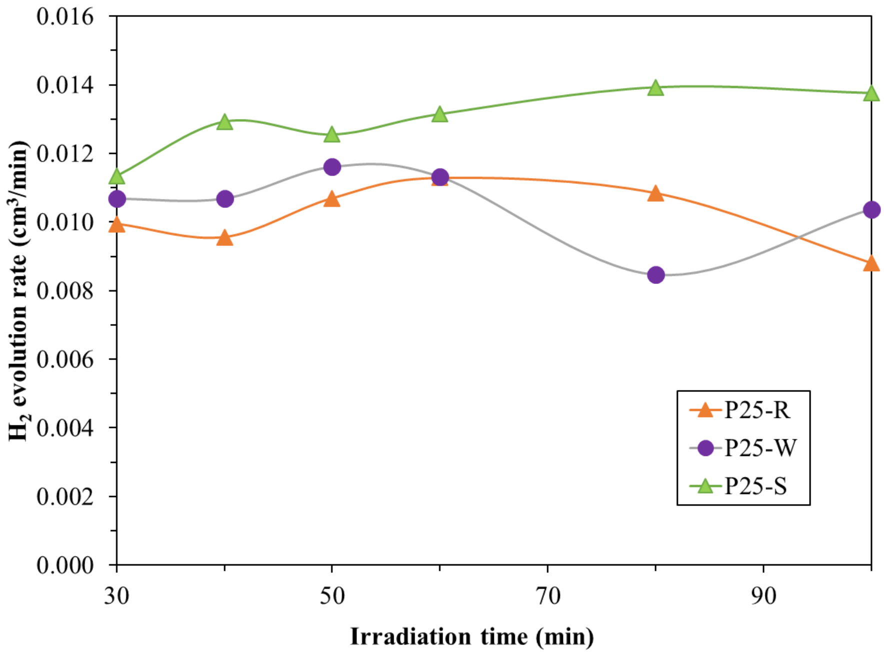

Figure S1. H_2 production rate using oxalic acid as sacrificial agent and selected P25-Au-based composites as photocatalysts.

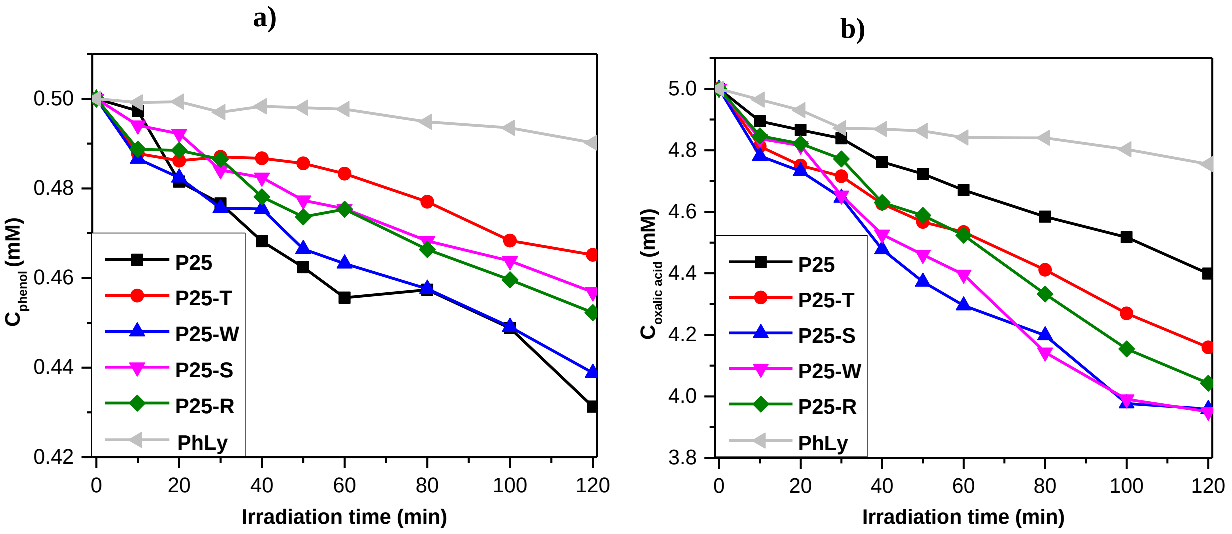

Figure S2. Degradation curves for phenol (**a**) and oxalic acid (**b**) of the prepared nanocomposites under Vis irradiation (PhLy refers to the degradation without photocatalyst).
